# Biosecurity measures and effects on health performance and antibiotic use in semi-intensive broiler farms in Uganda

**DOI:** 10.1016/j.onehlt.2025.101039

**Published:** 2025-04-15

**Authors:** Dreck Ayebare, Irene Mbatidde, Naomi P. Kemunto, Dishon M. Muloi, Eugine L. Ibayi, Søren Saxmose Nielsen, Dickson Ndoboli, Kristina Roesel, Bernd-Alois Tenhagen, Arshnee Moodley

**Affiliations:** a International Livestock Research Institute, Kampala, Uganda; b Department of Veterinary and Animal Sciences, University of Copenhagen, Copenhagen, Denmark; c Dahlem Research School of Biomedical Science, Department of Veterinary Medicine, Freie University, Berlin, Germany; d National Agricultural Research Organization, Mbarara, Uganda; e German Federal Institute of Risk Assessment (BfR), Berlin, Germany; f International Livestock Research Institute, Nairobi, Kenya; g Institute of Infection, Veterinary and Ecological Sciences, University of Liverpool, Liverpool, United Kingdom; h Department of Animal breeding and husbandry in the Tropics and Subtropics, University of Hohenheim, Stuttgart, Germany

**Keywords:** Prevention, Antibiotic use, Mortality, Vaccines, Poultry

## Abstract

**Background:**

Effective biosecurity measures prevent the spread of pathogens, thereby reducing the need for antibiotic use on livestock farms. However, quantitative data on these practices and health outcomes in semi-intensive broiler farms in low-income settings is limited. A longitudinal study in Wakiso, Uganda, aimed to evaluate biosecurity practices, health performance, and antibiotic use in such farms.

**Methods:**

The FarmUSE survey tool was used to collect data from 19 farms over two production cycles. A biosecurity risk assessment tool was used to quantify biosecurity. Blood samples (n = 342) were collected and analyzed using the ProFLOK® assay to assess antibody responses to vaccines for Newcastle disease (NCD), Infectious Bronchitis (IB), and Infectious bursal disease (IBD).

**Results:**

Median biosecurity scores were 26.3 % for external measures, 55.6 % for internal measures, and an overall score of 38.5 %. Sixteen farms reported respiratory signs, and 12 had gastrointestinal issues, with median mortality rates of 2.9 % in the first cycle and 4.6 % in the second. Antibiotic use was reported by 84 % and 77 % of farms in the first and second cycles, respectively. The most used antibiotics were tetracyclines, fluoroquinolones, and macrolides, with the highest usage occurring during the brooding phase. Good antibody responses were reported in only 10.5 % of flocks for NCD and 16 % for IBD, while all farms had poor responses against IB.

**Conclusion:**

Recurrent clinical signs could be attributed to insufficient biosecurity and inadequate vaccination outcomes, resulting in high antibiotic usage despite low mortality rates. Improving diagnostic access and strengthening the vaccine supply chain are essential. Identifying feasible and cost-effective biosecurity practices for semi-intensive broiler farms can enhance health outcomes, reduce antibiotic use, and boost productivity.

## 1 Introduction

The demand for animal-derived protein is projected to rise by 74 % to 114 % by 2050, fueled by global population growth [1]. Globally, poultry meat is expected to grow at the highest rate, at 121 %, with eggs following at 65 % [2]. In Uganda, the poultry sector is anticipated to grow to approximately 175 million birds by 2050, with consumption expected to increase by 320 % and 240 % for chicken meat and eggs, respectively [3]. The increased antimicrobial use (AMU) in low- and middle-income countries (LMICs) is primarily attributed to rising incomes, which result in increased demand for animal proteins and the intensification of livestock production to meet this demand. Agriculture intensification is estimated to increase AMU to approximately 104,000 tons by 2030 [4]. This trend is particularly significant in the urban and peri-urban areas of LMICs, where the commercialization of poultry production is on the rise due to high demand for animal-source protein. Nonetheless, the growth of poultry production faces challenges from infectious diseases, particularly viral infections, which are a significant threat to poultry production in Africa [5]. Due to the susceptibility of smallholder poultry production to these diseases, mortality rates can reach 50 % and 75 % in general flocks and brooding flocks, respectively [6]. Infectious diseases lead to production and economic losses and reduce food and nutrition security [7].

Effective biosecurity practices are essential in livestock production as they minimize the risk of pathogen introduction and spread both within and between farms [8,9]. Therefore, biosecurity can be critical in good animal health management and is a prerequisite to limiting AMU [10,11]. Poultry biosecurity assessments are typically conducted using questionnaires or biosecurity checklists [12,13]. The assessment of both internal and external biosecurity can be quantitative [12,14] or qualitative [15] depending on the structure and weighting of the different questions. Previous studies have demonstrated that questionnaires and checklists serve as practical tools to gather data on biosecurity and evaluate the compliance of poultry farms in Europe [8,16,17] and other settings in non-European countries [14,18,19], thereby identifying potential weaknesses and areas for intervention. The quantitative evaluation of biosecurity in semi-intensive poultry farms in Ethiopia [20] and Bangladesh [21] revealed low biosecurity scores. On the contrary, studies in several European countries, such as Belgium, Spain, and the Netherlands [22,23], as well as in the Philippines [14] reported high biosecurity scores; however, these poultry farms operated under an intensive production system. This highlights the differences in implementing biosecurity practices across different poultry production intensities.

Uganda has developed guidelines for infection prevention and appropriate AMU in the poultry sector [24], which promote good management practices on poultry farms and highlight biosecurity as a critical tool to achieve desired outcomes. However, more quantitative data on biosecurity implementation in semi-intensive farms, health performance, and AMU are needed to inform interventions to promote productivity and reduce AMU. Therefore, this study aimed to evaluate the levels of biosecurity, mortality, morbidity, vaccine efficacy, and antibiotic use in semi-intensive broiler farms in Wakiso district, Uganda.

## 2 Materials and methods

### 2.1 Study location and design

A longitudinal study was conducted on 19 broiler farms selected from a 2021 cross-sectional survey of 202 farms in Wakiso district, central Uganda. The initial survey showed that 86 % of farms reared 200–1000 birds under an intensive production system [25]. To ensure homogeneity in production scale and purpose of the chicken, farms were purposively selected based on this production range. Additionally, only farms that demonstrated a willingness to participate were included. This sampling strategy minimized variability, improved the feasibility of longitudinal follow-up, and allowed for a focused assessment of biosecurity, mortality, morbidity, vaccine efficacy, and antibiotic use within these farms.

**Fig. 1:**
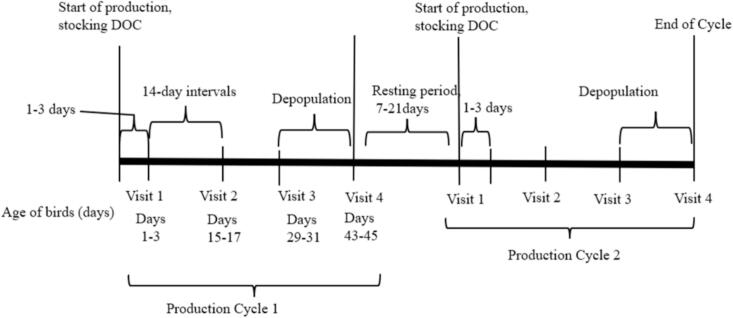
Illustration of the longitudinal study design. DOC is day-old chicks.

Wakiso district is a significant area for poultry farming, with a chicken population of approximately 5.6 million birds [26]. Chickens are mainly raised on a semi-intensive production scale (specifically, chickens kept indoors in flock sizes of 100–1000 birds) and an intensive production scale (farms with more than 1000 chickens). Selected farms were monitored for two complete production cycles, with four visits conducted every 2 weeks within each cycle to collect data (Fig. 1). The initial visit occurred within 1–3 days following the purchase of day-old chicks, and the last visit was immediately after depopulation (Days 43–45).

Twenty farms were initially recruited; however, one farm did not complete the first cycle, as the study team was unable to access the farm premises during the third visit and was therefore excluded from the analysis. Six farms that had completed the first cycle were excluded from the second cycle because they were unable to restock during the study period; therefore, the second cycle consisted of only 13 farms. The initial visit of the first cycle gathered baseline information on farm demographics, biosecurity measures, the demographics of the farmers, and antibiotic use practices from previous cycles. Although information on the current flock, such as the date of stocking and the number of day-old chicks, was captured during the first visit, data on mortality, clinical signs, and antibiotic use were not recorded. Subsequent visits were conducted to obtain data on mortality, morbidity, disease symptoms, antibiotic use, and vaccination practices.

### 2.2 Data collection

We used the FarmUSE survey tool [27] to gather baseline and follow-up information about farmers and farm demographics, management practices (including biosecurity and vaccination), production parameters, health parameters, and antibiotic use on the first visit to the farms. Data captured during the follow-up visits included antibiotic use, vaccinations, clinical signs, and mortality. Research assistants were trained in how to administer the questionnaires. The farmer reported data on mortality and morbidity, while the researchers collected information on biosecurity measures through observation and responses to the survey questions [27]. The completed questionnaires were uploaded onto the Open Data Kit (ODK) software [28].

Data was collected between May and November 2023. Responses to biosecurity questions were weighted using the poultry biosecurity assessment tool [29]. Specifically, the biosecurity section consisted of ten sub-components. These, in turn, were grouped as internal or external biosecurity. External biosecurity included seven components: 1) purchase of day-old chicks, 2) depopulation of broilers, 3) feed and water supply, 4) removal of manure and carcasses, 5) entry of visitors and staff, 6) infrastructure and biological vectors, and 7) location of the farm. Internal biosecurity consisted of three components: 1) disease management, 2) cleaning and disinfection, and 3) materials and measures between poultry houses.

### 2.3 Quantification of biosecurity

We utilized a flexible risk assessment tool designed to evaluate and weight biosecurity practices on small- and medium-scale poultry farms in LMICs. Scores were assigned to various biosecurity components as described in the tool [29]. Cumulative scores were obtained for each component: external, internal, and overall biosecurity for each farm. To compare biosecurity components on each farm, the maximum score allocated for each component was 72.5, comprising 27 points for internal biosecurity and 45.5 points for external biosecurity.

### 2.4 Assessment of vaccine efficacy

The efficacy of Newcastle disease (NCD), Infectious bronchitis (IB), and Infectious bursal disease (IBD) vaccines was assessed by measuring their ability to stimulate an immune response that provides adequate protection against NCD, IB, and IBD, respectively. The kit supplier recommends a sample size of 18 birds per flock of 500 to 1000 birds. Based on an expected vaccine efficacy of 95 % (with a precision of +/− 10 % at the 2-sided 5 % significance level), 342 birds were sampled in the 19 farms.

Serum samples (n = 342) were collected from 18 randomly selected birds per flock during the third visit of the first production cycle. The collected sera were analyzed using the ProFLOK® indirect ELISA test for the Newcastle disease virus [30], Infectious bronchitis virus [31], and Infectious bursal disease virus [32] to determine the antibody levels against each virus. Data on vaccination dates, administration routes, and personnel involved in vaccination were obtained through the questionnaire. The mean antibody titers (MT), geometric mean titers (GMT), and coefficient of variation (CV) were calculated for each flock. Following the manufacturer's guidelines, users are advised to define “protective” titer targets based on their flocks' specific health and productivity metrics. For this study, we adopted target values established by the RTC laboratory at COVAB, Makerere University, for broiler flocks reared in Uganda. The laboratory's test protocol outlines the expected normal antibody titres and CV for broilers vaccinated against each disease. These values are summarized in Table 1, Table 2.

**Table 1:** Normal range for ProFLOK® target mean antibody titres for broilers against each vaccine.

Type of vaccine/agent	Expected normal range of mean titer values
Infectious bronchitis virus	5000–10,000
Newcastle disease virus	3000–9000
Infectious bursal disease virus	4000–7000

**Table 2:** Coefficient of variation interpretation. The uniformity of the response is expressed as the Coefficient of Variation (CV). The lower the CV, the more uniform the flock's protection against infection.

Coefficient of variation (CV)	CV interpretation
0–10	Excellent
11–30	Good
30–50	Fair
>50	Poor

Farms were classified into three categories based on their mean titers and the CV:

1. Farms with mean titers that fall within the normal ranges and exhibit a fair, good, or excellent CV were considered to have good vaccination outcomes.
2. Farms with mean titers within normal ranges but with a poor coefficient of variation (CV) were considered to have fair vaccination outcomes.
3. Farms with mean titers below the normal range and a poor coefficient of variation (CV) were considered to have poor vaccination outcomes.

### 2.5 Quantification of mortality

The number of birds that died between visits was recorded at each visit. The cumulative mortality rate for each production cycle was then calculated as the ratio of the total number of deaths recorded to the number of birds stocked at the start of the cycle.

### 2.6 Morbidity

Clinical manifestations (e.g., sneezing, coughing, diarrhea, lameness, inappetence, etc.) noted in the flock were documented during visits 2 and 3. Depending on the bird's affected body system, clinical signs were classified into four categories: digestive, respiratory, musculoskeletal, or nervous.

### 2.7 Antibiotic use

Antimicrobial use was quantified using treatment incidence (TI), a standardized metric that estimates the number of defined daily doses administered per animal during a given period. TI was calculated using the formula:

$$\mathrm{TI}=\text{Total milligrams of active ingredient administered}/\left(\text{DDDvet}\times \text{average body weight}\;\left(\mathrm{kg}\right)\times \text{number of treated birds}\right).$$

The total quantity of each active ingredient administered was determined by multiplying the volume of the product given by its concentration. Defined daily doses (DDD) for broilers were obtained from international guidelines established by the European Medicines Agency (EMA) [33].

To account for body weight, average bird weights were estimated by production phase using standard growth patterns of broilers. In the absence of localized national standards, these estimates were based on typical broiler performance in Uganda and comparable production systems. Broilers were assumed to weigh approximately 0.2–0.5 kg during the brooding phase, 0.9–1.4 kg in the growing phase, and 2.0–2.5 kg in the finishing phase, consistent with global benchmarks and data from Ugandan extension manuals and performance studies [34,35].

### 2.8 Data and statistical analysis

Due to the small number of farms included in the study, only descriptive statistics were performed. Respondent characteristics were summarized using frequencies, while biosecurity measures were illustrated with box-and-whisker plots for the biosecurity categories (internal and external) and their components. Furthermore, descriptive statistics, including medians and interquartile ranges, were calculated for each biosecurity component. Similarly, mortality rates per visit were illustrated using box-and-whisker plots. Antimicrobial use was determined based on treatment incidence, while morbidity was reported based on frequencies of occurrence. Vaccine administration and vaccination outcomes were also presented using frequencies and proportions.

R software [36] was used to manipulate data, run descriptive statistics, and generate plots and tables.

## 3 Results

### 3.1 Respondent characteristics

Among the 19 farm owners or managers interviewed, 63 % were male (Table 3). Only 32 % of the respondents had formal training in poultry production, and 5.3 % had less than five years of experience in poultry farming. Additionally, 42 % of the participants fell within the age range of 36 to 55.

**Table 3:** Characteristics of respondents in the study farms.

Characteristic	N = 19 (%)
Sex	
Female	7 (37 %)
Male	12 (63 %)
Respondent type	
Manager	6 (32 %)
Owner	13 (68 %)
Education level	
Primary	5 (26 %)
Secondary	7 (37 %)
Tertiary	7 (37 %)
Age	
>55	4 (21 %)
20–35	7 (37 %)
36–55	8 (42 %)
Experience of the respondent	
<1 yrs	1 (5.3 %)
1–5 yrs	13 (68.4 %)
5–10 yrs	1 (5.3 %)
>10 yrs	4 (21 %)
Formal training in poultry management	
None	13 (68 %)
Present	6 (32 %)

### 3.2 Biosecurity

**Fig. 2:**
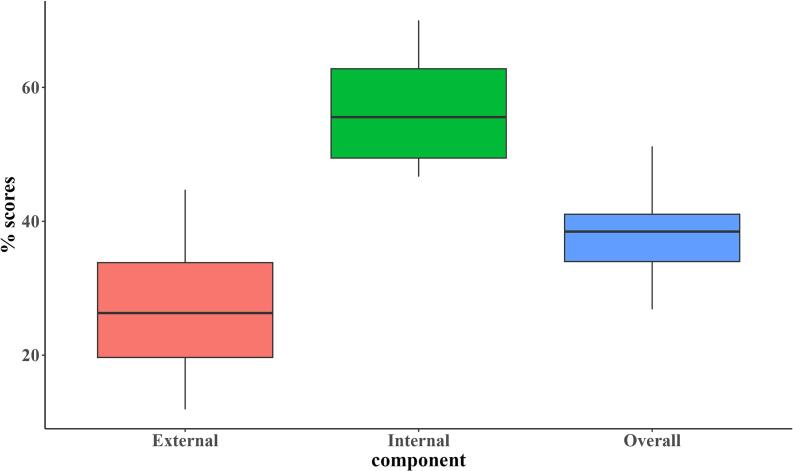
The distribution of biosecurity scores for internal, external, and overall biosecurity across 19 poultry farms. Each score represents a percentage of that category's maximum possible biosecurity score. The boxes illustrate the interquartile ranges, while the horizontal line denotes the median score for each category.

The median overall biosecurity score was 38.5 % (IQR = 34.0–41.0 %) (Fig. 2). Two farms had scores >50 % (51 % and 52 %). The median score for internal biosecurity was 55.6 % (IQR = 49.4–62.8 %), and the score for external biosecurity was 26.3 % (IQR = 19.7–33.8 %).

**Fig. 3:**
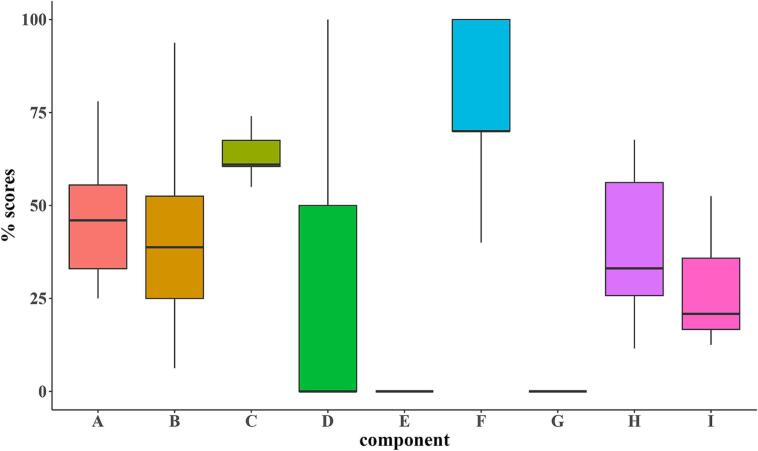
Distribution of biosecurity scores across different sections at poultry farms. Each section's score is presented as a percentage of the maximum score for that section. The boxes illustrate the interquartile ranges, while the horizontal line denotes the median score for each component.

Among the components assessed, practices related to infrastructure and biological factors (**A**), including housing, fencing, and access to coops by wild birds and pets, had a median score of 46 % (IQR = 33–55.5 %). Feed and water supply practices (**B**), including water source and treatment and feed source and storage, had a median score of 38.8 % (IQR = 25–52.5 %). Disease management practices (**C**), including vaccination strategies, stocking density, and management of sick birds, had a median score of 61 % (IQR = 60.5–67.5 %). Farm location (**D**) had a median score of 0 (IQR = 50 %), while practices associated with purchasing one-day-old chicks (E) had a mean score of 0 (IQR = 0). The movement of materials and measures between compartments (**F**) had a mean score of 70 % (IQR = 70–100 %). Practices related to removing dead animals and manure (**G**) had a mean score of 0 (IQR = 0). Cleaning and disinfection of farm facilities (**H**) had a mean score of 33.1 % (IQR = 25.8–56.2 %), while practices related to visitors and personnel entry (**I**) had a mean score of 20.8 % (IQR = 16.7–35.8 %). These results, including the range between farms, are illustrated in Fig. 3.

### 3.3 Clinical signs

All farms reported at least one occurrence of clinical signs in their flocks during the study. Clinical signs were reported in 17/19 farms in the first cycle, while 11/13 reported clinical signs in the second production cycle (Table 4). Respiratory signs were the commonly reported group of signs [16], followed by digestive signs [12]. Musculoskeletal signs were reported seven times, while nervous signs were reported two times.

**Table 4:** Frequency of clinical signs reported in the two production cycles.

Clinical signs (number of farms)				
	Digestive (n^a^= 12)	Musculoskeletal (n = 7)	Nervous (n = 2)	Respiratory (n = 16)
Cycle1-visit1	0	0	0	0
Cycle1-visit2	3 (25 %)	2 (29 %)	0	6 (38 %)
Cycle1-visit3	4 (33 %)	3 (43 %)	0	5 (31 %)
Cycle2-visit1	2 (17 %)	1 (14 %)	0	2 (13 %)
Cycle2-visit2	2 (17 %)	1 (14 %)	1 (50 %)	1 (6.3 %)
Cycle2-visit3	1 (8.3 %)	0	1 (50 %)	2 (13 %)

a n: the number of farms reporting a given clinical sign.

### 3.4 Mortality

**Fig. 4:**
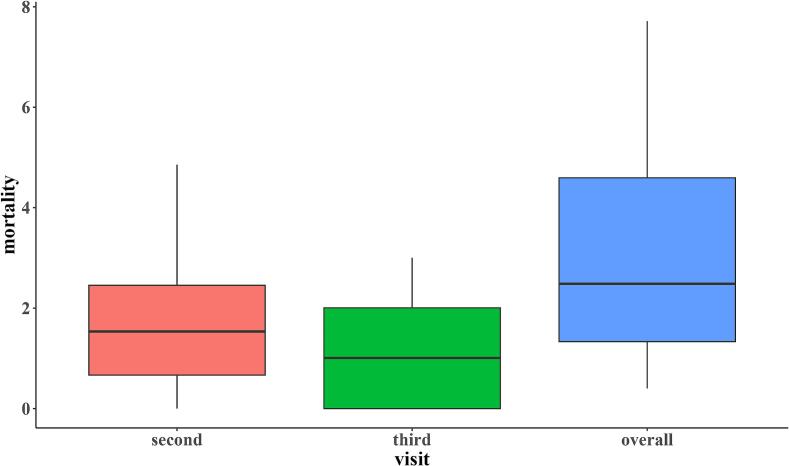
Mortality rates in the first production cycle in 19 semi-intensive broiler farms. The boxes illustrate the interquartile ranges, while the horizontal line denotes the median mortality for each visit.

Fig. 4 shows the mortality rates for all farms between visits 1–3 in the two cycles. The median overall mortality was 2.5 % (IQR = 1.3–4.6 %) in the first cycle for 19 farms. Within the first cycle, at the second visit, the median mortality was 1.5 % (IQR = 0.7–2.5 %), and at the third visit, it was 1.0 % (IQR = 0–2.0 %).

**Fig. 5:**
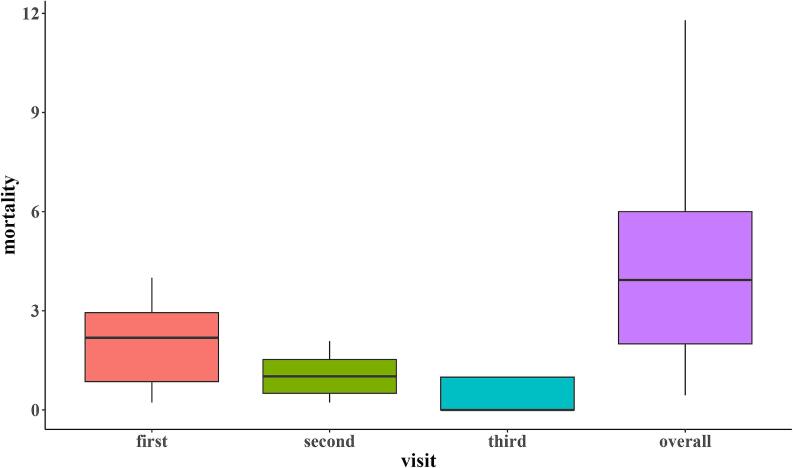
Mortality rates in the second production cycle in 13 semi-intensive broiler farms. The boxes illustrate the interquartile ranges, while the horizontal line denotes the median mortality for each visit.

In the second cycle, the median mortality was 3.9 % (IQR = 2–4 %) (Fig. 5). During the first visit, the median mortality was 2.2 % (IQR = 0.9–2.9 %). The median mortality was 1.0 (IQR = 0.5–1.5 %) on the second visit and 0 (IQR = 0–1.0 %) during the third visit.

### 3.5 Antibiotic use patterns

Of the 19 farms surveyed in the first production cycle, 84 % (16 farms) reported using antibiotics, while 77 % (10/13 farms) reportedly used antibiotics during the second cycle (Table 5).

**Table 5:** Antibiotic usage on farms during the different visits of the two production cycles.

	First cycle		Second cycle		
Visit	2nd	3rd	1st	2nd	3rd
Antibiotic use on the farm	12/19 (63 %)	11/19 (58 %)	4/13 (31 %)	6/13 (46 %)	7/13 (54 %)
Used antibiotics when birds were sick	8/12 (67 %)	10/11 (91 %)	4/13 (31 %)	4/7(57 %)	5/7 (71 %)

**Fig. 6:**
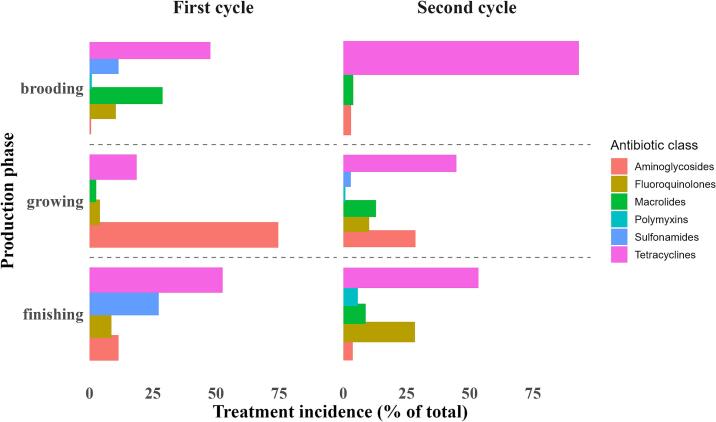
Relative contribution of antibiotic classes to treatment incidence throughout the two cycles. Bars represent the proportion of total treatment incidence attributed to each antibiotic class during the brooding, growing, and finishing phases, categorized by production cycle.

In the first cycle, a higher proportion of the farms reported using antibiotics, with 63 % and 58 % reporting antibiotic use in the second and third visits, respectively. In the second cycle, 31 %, 46 %, and 54 % reported use at the first, second, and third visits, respectively. Farmers mostly used antibiotics in both cycles when the birds were sick (Table 5). Fig. 6 illustrates the frequency of antibiotic use. Tetracyclines and macrolides were the most frequently used antibiotics in both production cycles.

### 3.6 Vaccination

All farms administered at least one IB, IBD, and NCD vaccine. Of the 19 farms in the first cycle, 53 % (10/19) administered a booster dose of NCD and IB in drinking water within one week after stocking. In contrast, 53 % and 26 % of farms administered additional boosters during the third and fourth weeks. For the IBD vaccine, 42 % (8/19) of farms provided it during the second week and 58 % (11/19) during the third week (Table 6).

**Table 6:** Percentage distribution of Newcastle disease, Infectious bronchitis, and Infectious bursal disease vaccine administration during the first production cycle.

	Week 1 (n = 19)	Week 2 (n = 19)	Week 3 (n = 19)	Week 4 (n = 19)
ND	10 (53 %)	0	10 (53 %)	5 (26 %)
IB	10 (53 %)	0	10 (53 %)	5 (26 %)
IBD	0	8 (42 %)	11 (58 %)	0

**Fig. 7:**
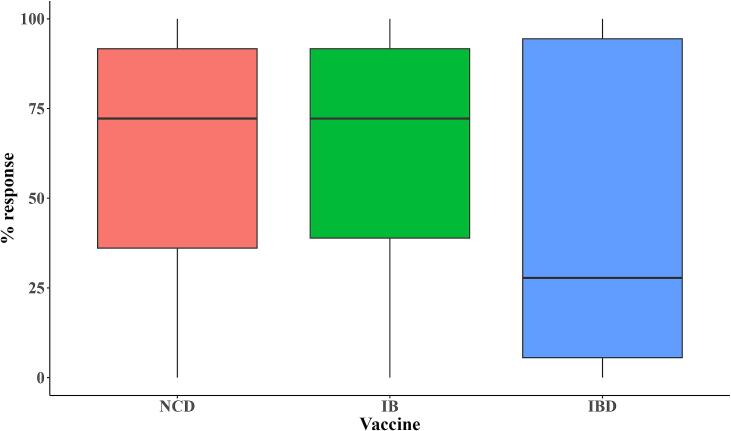
Distribution of percentage immune response to vaccines on farms. The boxes illustrate the interquartile ranges, while the horizontal line denotes the median percentage response for each vaccine.

The median percentage of birds with antibodies to the vaccines was 72.2 % for NCD (IQR = 36.1–91.7 %), 72.2 % (IQR = 38.9–91.7 %) for IB, and 27.8 % (IQR = 5.6–94.4 %) for IBD (Fig. 7). NCD and IB showed a higher and more consistent response than IBD, which was more variable.

Although all farms reported administering vaccines to their flocks, only 43–64 % of tested birds had antibodies against the vaccinated diseases. Strong antibody responses were observed in only two farms for the NCD vaccine and three for the IBD vaccine (Table 7). A fair vaccination outcome was observed in only one farm for IB, while poor vaccination outcomes were prevalent across all three vaccines.

**Table 7:** Vaccination outcomes for the three vaccines administered to chickens on the study farms during the first production cycle.

Category	Newcastle disease	Infectious bronchitis	Infectious bursal disease
Good vaccination	10.5 % (2/19)	0	16 % (3/19)
Fair vaccination	0	5 % (1/19)	0
Poor vaccination	89.5 % (17/19)	95 % (18/19)	84 % (16/19)

## 4 Discussion

This study evaluated the implementation levels of biosecurity measures, health performance, and antibiotic use in semi-intensive broiler farms in Wakiso, Uganda. Most farms were found to have biosecurity scores with a median of less than 50 %. Generally, farms had higher scores for internal biosecurity (i.e., preventing the spread of pathogens within their premises) than for external biosecurity (i.e., preventing the introduction of pathogens). Similar findings have also been reported in commercial chicken farms in Ethiopia [37] and Uganda [38,39].

This could be due to the inadequate investment in physical infrastructure required to implement barriers and establish access protocols. As per the FAO report, semi-intensive poultry farms in Wakiso are small-scale enterprises, typically established with limited budgets and employing basic processes and production practices [40].

Additionally, limited knowledge of biosecurity practices was identified as the main reason for the low adoption of biosecurity measures in smallholder poultry farms in the Nyanza region of Kenya [41]. Poultry farmers often lack access to information and funding for the implementation of effective biosecurity measures [42]. This challenge is reflected in Uganda, where smallholder poultry farmers frequently encounter limited access to agricultural extension services and minimal financing for livestock-related investments [43]. These structural barriers not only hinder the adoption of external biosecurity measures but also limit the overall capacity of farms to implement preventive health measures. Several farms reported clinical signs related to the respiratory and digestive systems during the two production cycles. These signs (including coughing, sneezing, bloody diarrhea, inappetence, etc.) could be attributed to environmental factors such as poor ventilation, nutritional deficiencies, or infectious pathogens [44]. Despite frequently reporting these signs, mortality was low. This is in line with findings from a study in semi-intensive broiler farms in Vietnam, which also highlighted these respiratory and digestive signs prompting antibiotic use [45].

Despite regular antimicrobial use, persistent clinical signs could be linked to the misdiagnosis of diseases. This is usually a result of farmers treating without seeking professional advice. This practice has been reported in a study conducted in the western district of Masindi in Uganda, where approximately 45 % of semi-intensive farmers treated sick birds without seeking veterinary advice [46]. This practice helps manage secondary bacterial infections in the flock, thereby reducing overall mortality rates.

Consistent with our findings, studies have shown that broiler chickens experience the highest mortality during brooding [45,47], and mortality rates decrease as they age [48]. Key management-related factors influencing early-stage chick mortality include ventilation type, flock size, shipping distance, and delivery route [17,49]. However, in contrast to our findings, significantly higher crude mortality rates were reported in broiler farms lacking adequate biosecurity measures in Bangladesh [48]. The low mortality rates observed in our study may be attributed to the frequent use of antibiotics. Antibiotic use on farms was prevalent, even in flocks with no disease incidence, for disease prevention, a common practice in semi-intensive broiler farms. This practice was previously reported in Wakiso, where 42 % of farms administered antibiotics for prophylactic purposes, including the critically important antibiotics such as fluoroquinolones [25,50]. Frequent use of these antibiotic classes has also been reported in Bangladesh [51].

Tetracyclines and macrolides were the most frequently used classes of antibiotics, particularly during the brooding phase. This trend likely reflects standard prophylaxis and treatment for respiratory conditions in the early stages of growth. Their popularity is also linked to their affordability and widespread availability [10]. The extensive use of fluoroquinolones is concerning, given their classification by the WHO as highest priority critically important antimicrobials (HPCIAs) for human medicine [52]. While their use may be licensed and diagnostically appropriate due to their broad-spectrum efficacy, their widespread application raises important public health concerns related to antimicrobial resistance [53].

This pattern of antimicrobial use also seems to reflect broader gaps in disease prevention, including ineffective vaccination. Inadequate immunisation outcomes may be leading farmers to turn to antibiotics as a compensatory measure [45].

Vaccination outcomes for all three targeted poultry diseases were poor. Several factors could have influenced the effectiveness of vaccination, including the stability of the vaccine and its transportation, storage, and administration [54]. Agrovet shops often do not maintain the stable temperature controls necessary for proper vaccine storage [55], which can affect the potency and safety of the vaccines. Studies have shown that temperature significantly impacts vaccine efficacy [56,57]. Furthermore, a study in Uganda revealed that some drug stockists reconstitute vaccines for retail purposes while others sell expired medications to farmers by altering labels to misrepresent their shelf life [58]. The limited formal training in poultry management reported among these farmers may have impacted their ability to handle and administer vaccines properly on the farm. A study by Bosha and Nongo in poultry farms in Nigeria revealed that breaches in the administration, handling, and transportation of vaccines were responsible for vaccine failures [59].

The limited number of participating broiler farms resulted in a small dataset, allowing only descriptive statistical analysis. Nevertheless, this study represents a longitudinal, quantitative assessment of biosecurity, health performance, and antibiotic use across two production cycles, offering valuable insights despite the limitation in sample size.

The biosecurity measures implemented on these farms were generally inadequate, particularly in terms of external biosecurity. Although internal biosecurity practices were relatively stronger, the frequent occurrence of clinical signs and poor vaccination outcomes highlight areas for improvement. While mortality rates remained within acceptable limits, the reliance on antibiotics, especially for prophylactic use, was high. This could be due to fear of disease outbreaks. This is supported by a study on semi-intensive broiler farms in Vietnam, indicating that morbidity, rather than mortality, influences antimicrobial use [45].

Given the observed patterns of antibiotic class usage and timing, stewardship efforts should prioritise the early production phases. Reducing unnecessary antibiotic use in brooding, alongside improved diagnostic support, may help shift reliance away from habitual prophylaxis.

Although this study does not evaluate specific interventions, its findings suggest several potential areas for improvement. Strengthening external biosecurity through enhanced access control, fencing, and sanitation infrastructure is likely to reduce the introduction of pathogens. Additionally, improving vaccine handling, particularly by ensuring the reliability of the cold chain, could enhance vaccination success and reduce disease pressure. Furthermore, promoting responsible antimicrobial use can be achieved by improving access to diagnostic services.

In conclusion, this study highlights low implementation of biosecurity measures, poor vaccine efficacy, and high antimicrobial use in semi-intensive broiler production. The frequent use of tetracyclines and macrolides, particularly during brooding, along with the reported use of fluoroquinolones, highlights the need for integrated and preventive poultry health strategies. While further research is needed to identify context-specific, cost-effective interventions, the findings suggest practical entry points for reducing antibiotic use. These insights can guide future research and policymaking, ultimately contributing to more sustainable and responsible broiler production practices.

## CRediT authorship contribution statement

**Dreck Ayebare:** Writing – review & editing, Writing – original draft, Visualization, Validation, Project administration, Methodology, Investigation, Formal analysis, Conceptualization. **Irene Mbatidde:** Writing – review & editing, Writing – original draft, Visualization, Validation, Methodology, Investigation, Conceptualization. **Naomi P. Kemunto:** Writing – review & editing, Methodology, Formal analysis. **Dishon M. Muloi:** Writing – review & editing, Supervision, Project administration, Methodology, Investigation. **Eugine L. Ibayi:** Writing – review & editing, Methodology, Formal analysis. **Søren Saxmose Nielsen:** Writing – review & editing, Methodology, Formal analysis. **Dickson Ndoboli:** Writing – review & editing, Methodology, Investigation, Conceptualization. **Kristina Roesel:** Writing – review & editing, Supervision, Project administration, Investigation, Funding acquisition. **Bernd-Alois Tenhagen:** Writing – review & editing, Supervision, Methodology, Conceptualization. **Arshnee Moodley:** Writing – review & editing, Supervision, Funding acquisition, Conceptualization.

## Funding

This work was funded by the German Federal Ministry of Economic Cooperation and Development (BMZ) through the project “Boosting Uganda's Investment in Livestock Development” (BUILD). This study also received support from the CGIAR One Health initiative “Protecting Human Health Through a One Health Approach,” which was supported by contributors to the CGIAR Trust Fund (https://www.cgiar.org/funders/).

## Declaration of competing interest

The authors declare that they have no known competing financial interests or personal relationships that could have appeared to influence the work reported in this paper.

## Data Availability

Data will be made available on request.

## Appendix A Supplementary data

Supplementary material Image 1
