# Supplementary material for: Biosecurity measures and effects on health performance and antibiotic use in semi-intensive broiler farms in Uganda
Source: One Health. 2025 Apr 15;20:101039. doi: 10.1016/j.onehlt.2025.101039 (PMC12051712; doi:10.1016/j.onehlt.2025.101039)
Supplement: Supplementary file 1 — Supplementary material [file mmc1.docx]

**Detailed summary statistics for biosecurity measures, mortality, and vaccine responses**

Table A1: Biosecurity scores categorised by internal, external and overall biosecurity

| **Biosecurity category** | **Median (%)** | **IQR (%)** |
| --- | --- | --- |
| External | 26.3 | 19.7-33.8 |
| Internal | 55.6 | 49.4-62.8 |
| Overall | 38.5 | 34.0-41.0 |

Table A2: Biosecurity scores across different sections at poultry farms

| **Biosecurity component** | **Median (%)** | **IQR (%)** |
| --- | --- | --- |
| Infrastructure and biological factors **(A)** | 46 | 33-55.5 |
| Feed and water supply practices (**B**) | 38.7 | 25-52.5 |
| Disease management practices (**C**) | 61 | 60.5-67.5 |
| Farm location (**D**) | 0 | 0-50 |
| Purchasing one-day-old chicks (**E**) | 0 | 0 |
| movement of materials and measures between compartments (**F**) | 70 | 70-100 |
| Removal of dead animals and manure (**G**) | 0 | 0 |
| Cleaning and disinfection of farm facilities **(H)** | 33.1 | 25.8-56.2 |
| Visitors and personnel entry **(I)** | 20.8 | 16.7-35.8 |

Table A3: Mortality in the first production cycle

| **Visit** | **Median (%)** | **IQR (%)** |
| --- | --- | --- |
| Second | 1.5 | 0.7-2.5 |
| Third | 1.0 | 0-2.0 |
| Overall | 2.5 | 1.3-4.6 |

Table A4: Mortality in the second production cycle

| **Visit** | **Median (%)** | **IQR (%)** |
| --- | --- | --- |
| First | 2.2 | 0.9-2.9 |
| Second | 1.0 | 0.5-1.5 |
| Third | 0 | 0-1.0 |
| Overall | 3.9 | 2-6 |

Table A5: Distribution percentage immune response within flocks

| **Vaccine** | **Median (%)** | **IQR (%)** |
| --- | --- | --- |
| Newcastle disease virus | 72.2 | 36.1-91.7 |
| Infectious bronchitis virus | 72.2 | 38.9-91.7 |
| Infectious bursal disease virus | 27.8 | 5.56-94.4 |
